# The psychology of kink: A survey study investigating stigma and psychological mechanisms in BDSM

**DOI:** 10.1192/j.eurpsy.2022.2079

**Published:** 2022-09-01

**Authors:** A. Schuerwegen, M. Morrens, E. Wuyts, W. Huys, K. Goethals, I. De Zeeuw-Jans

**Affiliations:** 1 University Forensic Center (UFC), University Forensic Center (ufc), Edegem, Belgium; 2 Faculty of Medicine and Health Sciences, Collaborative Antwerp Psychiatric Research Institute (CAPRI), University Of Antwerp, Wilrijk, Belgium; 3 Collaborative Antwerp Psychiatric Research Institute (CAPRI), Faculty Of Medicine And Health Sciences, Campus Drie Eiken, University Of Antwerp, Edegem, Belgium; 4 Antwerp University Hospital, University Forensic Centre, Edegem, Belgium

**Keywords:** BDSM, Sensation seeking, stigma, coping

## Abstract

**Introduction:**

The past years BDSM (an acronym for bondage and discipline, dominance and submission, and sadism and masochism) has gained a significant amount of attention and popularity in the general population, portraying an inaccurate image of BDSM and the people who share these interests. Yet despite this increasing popularity, only little empirical research has focused on this subject and it’s possible driving mechanisms so far, sustaining the existing misconceptions and stigma towards BDSM in general and BDSM practitioners in specific.

**Objectives:**

We aimed to gain more insights on understanding the underlying psychological mechanisms, such as sensation seeking and coping, in people who participate in BDSM-related activities, as well as into the factors which contribute to the existing stigma and discrimination

**Methods:**

In a national survey study 256 Dutch-speaking BDSM-practitioners were compared to a matched sample of people from the general Belgian population (N = 300) who lack any interest in BDSM in two separate studies.

**Results:**

About 86% of the general population maintained stigmatizing beliefs about these sexual interests and practices. In regard to sensation seeking and coping, compared to controls, BDSM practitioners reported signifcantly higher levels of sensation seeking for all dimensions, as well as the use of more active coping skills.

**Conclusions:**

People who do not conform to the current social standards of our society often seem to remain the subject of stigmatization and discrimination. Further research is needed to explore the psychological processes that drive BDSM interests in order to destigmatize and normalize consensual BDSM-related activities.

**Disclosure:**

No significant relationships.

